# Stethoscope

**Published:** 1851-07

**Authors:** 


*Stethoscope.*—We desire to express our acknowledgments to Dr. Gooch, Editor of the Stethoscope, for his courtesy in transmitting, in anticipation of the issue of his Journal, a report of the proceedings of the Am. Med. Association. We had, however, already prepared the abstract contained in our last No., and we will defer farther notice of the subject until the appearance of the volume of Transactions.

